# Diagnosis of *Coxiella burnetii* Infection: Comparison of a Whole Blood Interferon-Gamma Production Assay and a *Coxiella* ELISPOT

**DOI:** 10.1371/journal.pone.0103749

**Published:** 2014-08-01

**Authors:** Teske Schoffelen, Gijs J. M. Limonard, Chantal P. Bleeker-Rovers, John J. M. Bouwman, Jos W. M. van der Meer, Marrigje Nabuurs-Franssen, Tom Sprong, Marcel van Deuren

**Affiliations:** 1 Department of Internal Medicine, Radboud university medical center, Nijmegen, The Netherlands; 2 Department of Pulmonary Diseases, Diakonessenhuis Utrecht, Utrecht, The Netherlands; 3 Institute for Life Sciences, University of Applied Sciences, Utrecht, The Netherlands; 4 Department of Medical Microbiology and Infectious Diseases, Canisius Wilhelmina Hospital, Nijmegen, The Netherlands; 5 Department of Internal Medicine, Canisius Wilhelmina Hospital, Nijmegen, The Netherlands; University of Cape Town, South Africa

## Abstract

Diagnosis of ongoing or past infection with *Coxiella burnetii*, the causative agent of Q fever, relies heavily on serology: the measurement of *C. burnetii*-specific antibodies, reflecting the host’s humoral immune response. However, cell-mediated immune responses play an important, probably even more relevant, role in infections caused by the intracellular *C. burnetii* bacterium. Recent studies have investigated interferon-gamma (IFN-γ) based assays, including a whole-blood IFN-γ production assay and a *Coxiella* enzyme-linked immunospot (*Coxiella* ELISPOT), as potential diagnostic tools for Q fever diagnosis. Both are in-house developed assays using stimulating antigens of different origin. The main objective of this study was to compare the test performance of the IFN-γ production assay and the *Coxiella* ELISPOT for detecting a cellular immune response to *C. burnetii* in Q fever patients, and to assess the correlation between both assays. To that end, both tests were performed in a well-defined patient group of chronic Q fever patients (n = 16) and a group of healthy seronegative individuals (n = 17). Among patients, both the Coxiella ELISPOT and the IFN-γ production assay detected positive response in 14/16. Among controls, none were positive in the Coxiella ELISPOT, whereas the IFN-γ production assay detected positive results in 1/17 and 3/17, when using Henzerling and Nine Mile as stimulating antigens, respectively. These results suggest the *Coxiella* ELISPOT has a somewhat higher specificity than the IFN-γ production assay when Nine Mile is used as antigen stimulus. The assays showed moderate correlation: the Spearman correlation coefficient *r* ranged between 0.37–0.60, depending on the antigens used. Further investigation of the diagnostic potential for *C. burnetii* infection of both assays is warranted.

## Introduction

Q fever is a zoonotic disease that occurs worldwide and is caused by the gram-negative, intracellular bacterium *Coxiella burnetii*. The Netherlands experienced a major Q fever outbreak between 2007 and 2010, with over 4,000 reported human infections [1]. Acute Q fever mostly presents as a flu-like illness, pneumonia or hepatitis, but initial infections can be asymptomatic in more than 50% of cases [2]. Chronic Q fever is a rare but serious complication that occurs in approximately 5% of all patients following the acute infection. This persisting infection typically presents with endocarditis or vascular infection, and has significant mortality rates, especially in case of diagnostic and therapeutic delay [3].

Evaluating chronic *C. burnetii* infection is challenging. Measurement of serum antibodies against *C. burnetii* is currently the ‘gold standard’ for Q fever diagnosis [3]. Diagnosis of chronic Q fever relies heavily on detection of high IgG antibody titers against phase 1 *C. burnetii*. This serological criterion is combined with PCR for *C. burnetii* DNA on blood or tissue (if available) and clinical assessment of any nidus of chronic infection [4], [5] .

Both humoral and cell-mediated immune responses are involved in the host’s immunity against the intracellular *C. burnetii* bacterium [6]–[8]. Therefore, it makes sense to explore the value of complementing conventional serology with an assessment of host specific cell-mediated immune responses to detect a chronic infection with *C. burnetii*. To this end, new immunological blood tests have been developed that are based on cellular immunity, measuring T-cell derived interferon-γ (IFN-γ) production in response to stimulation with *C. burnetii*. The first, the whole-blood IFN-γ production assay, was extensively investigated during a Q fever vaccination campaign and for the diagnosis of chronic Q fever [9]–[11]. The second, the *Coxiella* enzyme-linked immunospot (*Coxiella* ELISPOT), was explored in a small series of patients with past or chronic Q fever [12]. The IFN-γ production assay measures the amount of *C. burnetii* specific IFN-γ production, while the *Coxiella* ELISPOT measures the number of *C. burnetii* specific IFN-γ producing cells. These IFN-γ based assays are not yet routinely used for Q fever diagnosis. Both are in-house assays, using different stimulating antigens. The IFN-γ production assay uses in-house cultured Nine Mile phase 1 and the Q-vax vaccine containing Henzerling phase 1, while the *Coxiella* ELISPOT uses commercially available Nine Mile phase 1 and phase 2 antigens.

In the aftermath of the Dutch Q fever outbreak, we had the opportunity to use both IFN-γ based tests in parallel in a group of well-defined chronic Q fever patients. Volunteers with no history of Q fever and with negative Q fever serology served as a control group.

The purpose of this study was to compare the test performance (sensitivity and specificity) of the IFN-γ production assay and the *Coxiella* ELISPOT for detecting a cellular immune response to *C. burnetii* in Q fever patients and to determine the correlation between the assays.

## Materials and Methods

### Patients and control subjects

Chronic Q fever patients (n = 16) were recruited from the outpatient clinics of the participating hospitals. All fulfilled the criteria for probable (n = 4) or proven (n = 12, of which 3 Q fever endocarditis and 9 Q fever vascular infection) chronic Q fever according to the Dutch consensus statements on chronic Q fever [4]. Fourteen of the 16 patients were on long-term antibiotic treatment at inclusion. The median duration of antibiotic treatment at the time of blood collection was 21 months (range 0–59 months). The control individuals (n = 17) were similar with respect gender, somewhat younger, but had no history of Q fever and had negative Q fever serology as measured by immunofluorescence assay (Focus Diagnostics, Cypress, CA, USA) (Table 1). Informed consent was obtained from all subjects before blood donation and the study was approved by the local ethics committee (CMO regio Arnhem-Nijmegen).

**Table 1:** Characteristics of chronic Q fever patients and control individuals.

	Patients (n = 16)	Controls (n = 17)
**Age in yrs, median (range)**	68 (31–80)	46 (25–64)
**Males, number (%)**	13 (81)	14 (82)
**IgG anti-phase I titer** a **, median (range)**	2048 (128–32768)	<16 (n.a.)
**IgG anti-phase II titer** a **, median (range)**	3072 (256–32768)	<16 (n.a.)
**Duration of antibiotic treatment in months** a **, median (range)**	21 (0–59)	n.a.

n.a., not applicable.

a At the time of bloodsampling.

### IFN-γ production assay

The IFN-γ production assay was performed as previously described [9]. In short, heparinized whole blood was aliquoted into four separate 1.5 mL tubes at 0.5 mL per tube. The tubes were inoculated with either 10 µg/mL PHA (Sigma, St. Louis, MO), 100 ng/ml Q vax vaccine (see below), 10∧7/mL heat-inactivated *C. burnetii* Nine Mile phase I (see below), or nothing. The tubes were incubated in-vitro for 24 hours at 37°C and 5% CO_2_. The IFN-γ production was measured in the supernatant by ELISA (Pelikine compact, Sanquin, Amsterdam). Net IFN-response was expressed as the concentration of IFN-γ in the stimulated sample minus that in the unstimulated sample.

The Q-vax vaccine (CSL Biotherapies, Vic., Australia), contains formalin-inactivated whole cell phase 1 Henzerling strain.

Heat-inactivated phase 1 *C. burnetii* Nine Mile (NMI, RSA493) was kindly provided by H.I. Roest (Central Veterinary Institute, Lelystad, the Netherlands). Details about culture and preparation of this stimulating antigen are described elsewhere [9].

### Coxiella ELISPOT

The *Coxiella* ELISPOT was performed on peripheral blood mononuclear cells (PBMCs) isolated from heparinized blood and stimulated for 16–20 hours, as described before [12].

In precoated wells of PVDF strip plates (ELISpotpro; Mabtech) 100 µL of mononuclear cells were seeded at a density of 250,000 cells per well, and incubated with 50 µL of antigens, PHA (2.5 µg/mL) or nothing. As stimulating antigens, commercially available formalin-inactivated phase 1 and phase 2 Nine Mile (NMI and NMII, Virion-Serion Immunodiagnostica GmbH, Würzburg, Germany) were used. After incubation, the resulting number of spots, representing the number of individual T-cells producing IFN-γ following stimulation with *C. burnetii* antigens, were detected and enumerated using an ELISpot reader (Auto Immun Diagnostika GmbH, Strassberg, Germany).

### Statistics

Statistical analysis was performed using GraphPad Prism 5. Median (± interquartile range) IFN-γ production and number of spots were compared between groups using Mann-Whitney *U*-tests. Receiver Operating Characteristics (ROC) curves analysis were used to derive a cutoff for positivity of either assay. The cutoff was determined from the ROC curve by choosing the value that yielded empirical optimal sensitivity and specificity.

Correlation was reported by calculating the Spearman’s *r* with 95% Confidence Interval.

## Results and Discussion

**Figure 1:**
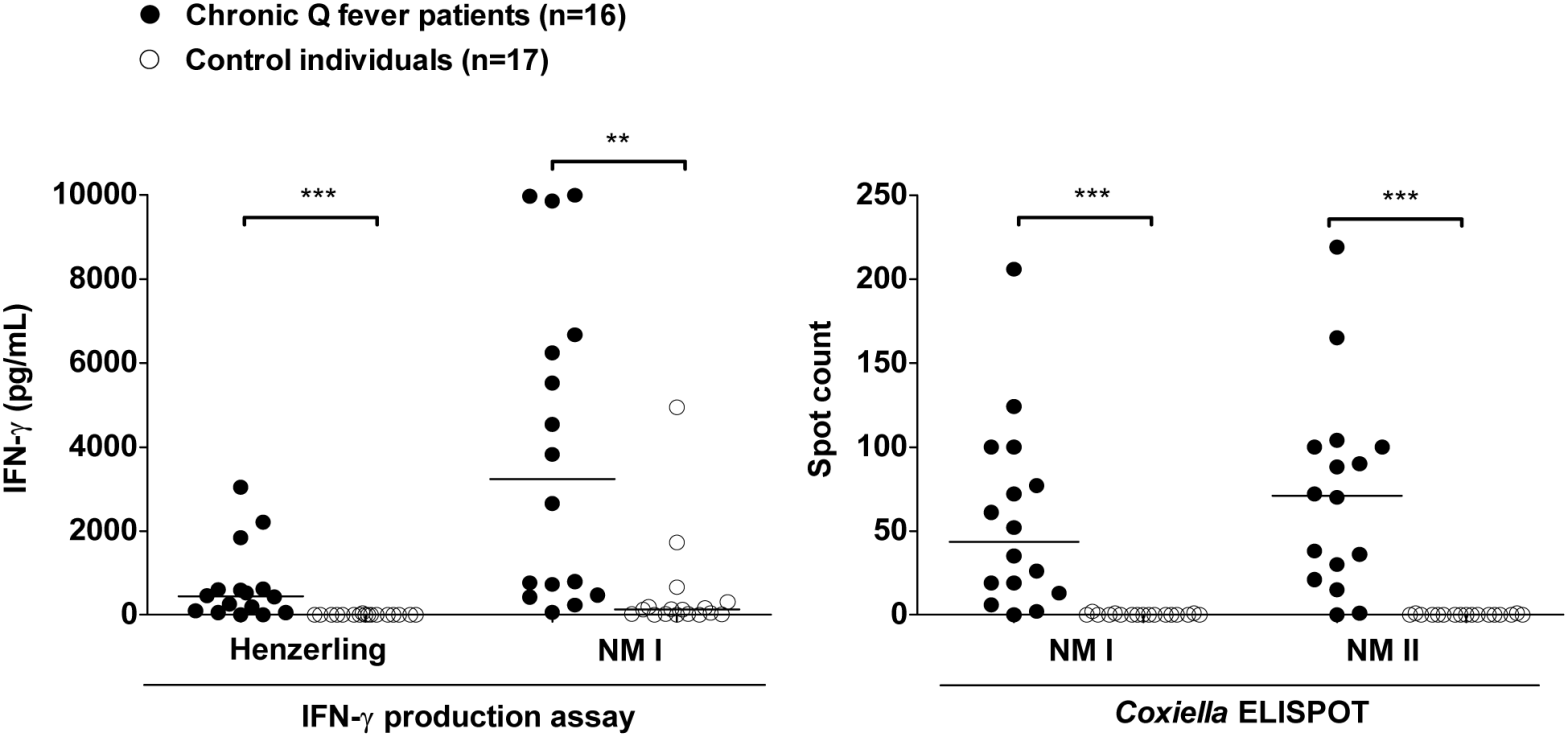
Results of the IFN-γ production assay and *Coxiella* ELISPOT in chronic Q fever patients and control subjects. *In vitro* IFN-γ production by whole blood in response to Henzerling and Nine Mile phase 1 antigens was measured in the IFN-γ production assay. The number of IFN-γ positive cells in response to Nine Mile phase 1 and Nine Mile phase 2 antigens was measured in the *Coxiella* ELISPOT. Individual values of patients and controls are shown separately, and the lines indicate the medians. Patients and controls were compared using the Mann-Whitney *U*-test. ***P<0.001, **P<0.01. Abbreviations: NMI, Nine Mile phase 1; NMII, Nine Mile phase 2; IFN-γ, interferon-gamma.

The amount of IFN-γ as measured in the IFN-γ production assay and the spot count obtained in the *Coxiella* ELISPOT were evaluated. The individual values are shown in Figure 1 for patients and control subjects separately. The median IFN-γ response to the Henzerling antigen in the patients was 440 pg/mL (IQR 62–612 pg/mL), whereas in control subjects, this was 0 pg/mL (IQR 0–0 pg/mL). The median IFN-γ response to the NMI antigen in the patients was 3238 pg/mL (IQR 534–6564 pg/mL), whereas in control subjects, this was 126 pg/mL (IQR 14–250 pg/mL). The median ELISPOT spot count after NMI stimulation was 44 (IQR 15–94) in patients and 0 (IQR 0–0) in controls, and after NMII stimulation 71 (IQR 23–100) in patients and 0 (IQR 0–0) in controls. In both assays, the differences between patients and control subjects were significant for each stimulating antigen. All of the samples tested responded to PHA mitogen.

**Figure 2:**
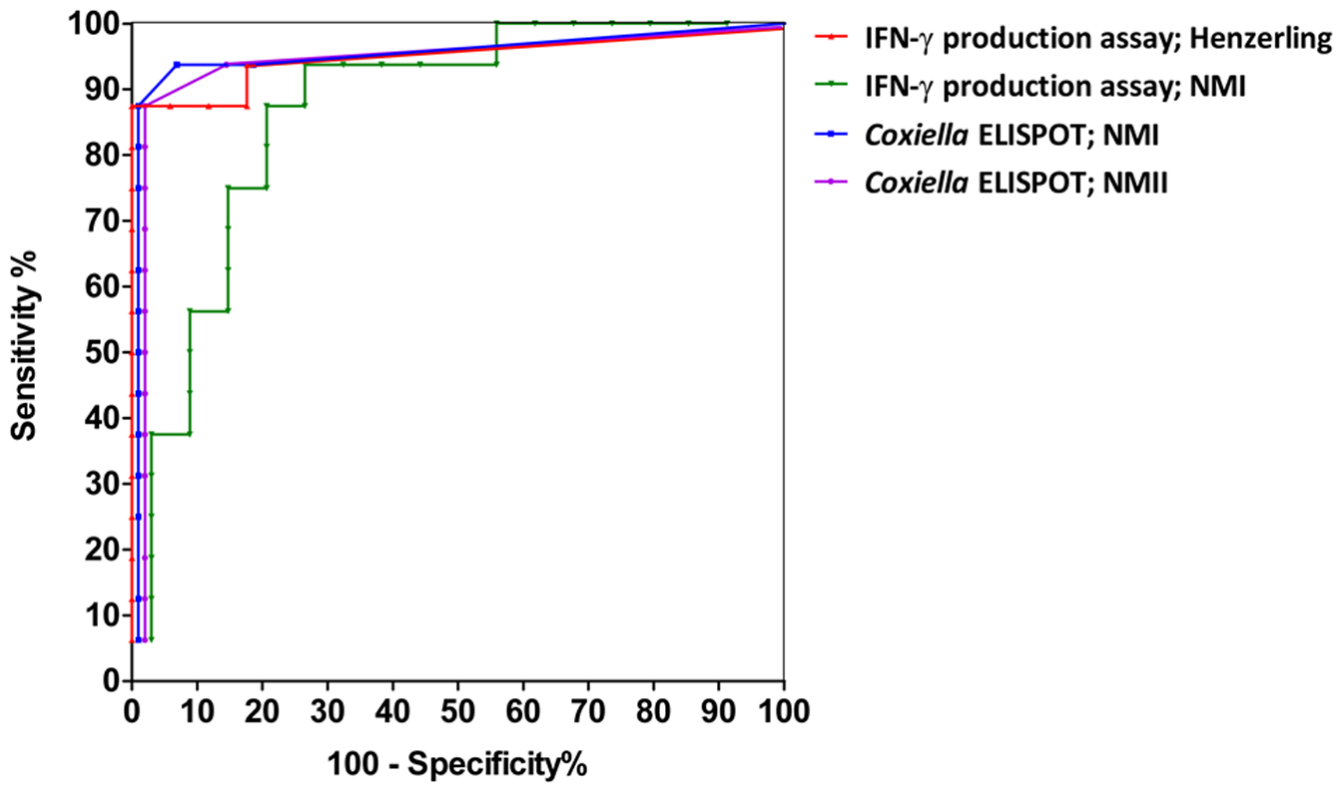
ROC curves of the IFN-γ production assay and the *Coxiella* ELISPOT. ROC curves are shown for each assay with each stimulating antigen separately. Abbreviations: NMI, Nine Mile phase 1; NMII, Nine Mile phase 2; IFN-γ, interferon-gamma.

To establish a cutoff for a positive response in both assays for each of the antigens, ROC curves were constructed (Figure 2). Cutoffs were derived from ROC curve analysis to yield empirical optimal sensitivity and specificity. For the IFN-γ production assay, this resulted in a cutoff of 45 pg/mL or 365 pg/mL when using the Henzerling or the NMI as stimulating antigen respectively. For the *Coxiella* ELISPOT, this resulted in a cutoff of 5 spots to both NMI and NMII. Given these cutoffs, the IFN-γ production assay detected positive response in 14/16 and the *Coxiella* ELISPOT was positive in 14/16 among the patients. Among the controls, the IFN-γ production assay detected positive response in 1/17 and 3/17, when using Henzerling and Nine Mile as stimulating antigens respectively, whereas none were positive in the *Coxiella* ELISPOT. These results suggest that the assays have comparable sensitivity in this population, but the *Coxiella* ELISPOT has a higher specificity than the IFN-γ production assay with Nine Mile as an antigen stimulus. This might be due to different antigens being exposed after heat-inactivation of the NMI strain as compared to the antigens present on the formalin-inactivated bacteria.

Furthermore, it is very well possible that the IFN-gamma production assay and the *Coxiella* ELISPOT assay do not measure the same features of the immune response. Although both tests measure *Coxiella* specific IFN-gamma production, the *Coxiella* ELISPOT assay singles out and quantifies the T lymphocyte compartment, whereas whole blood stimulation also includes blood IFN-gamma producing NK cells, reflecting also the innate part of the immune response. However, true positives in the control group cannot be excluded which in turn may underestimate specificity. A seroprevalence study in the Q fever epidemic area among patients with a valvular risk factor for chronic Q fever found 20.4% of the people to be seropositive for *C. burnetii* [13]. As the control subjects lived close to or in the epidemic area, although not having a Q fever history or detectable anti-*C. burnetii* antibodies, it is not unthinkable that any of the control subject could have a past exposure to *C. burnetii* that was not picked up by serology. Of note, two of the three control subjects that produced substantial amounts of IFN-γ after NMI antigen stimulation, also showed minimal response below the cutoff in the *Coxiella* ELISPOT.

**Figure 3:**
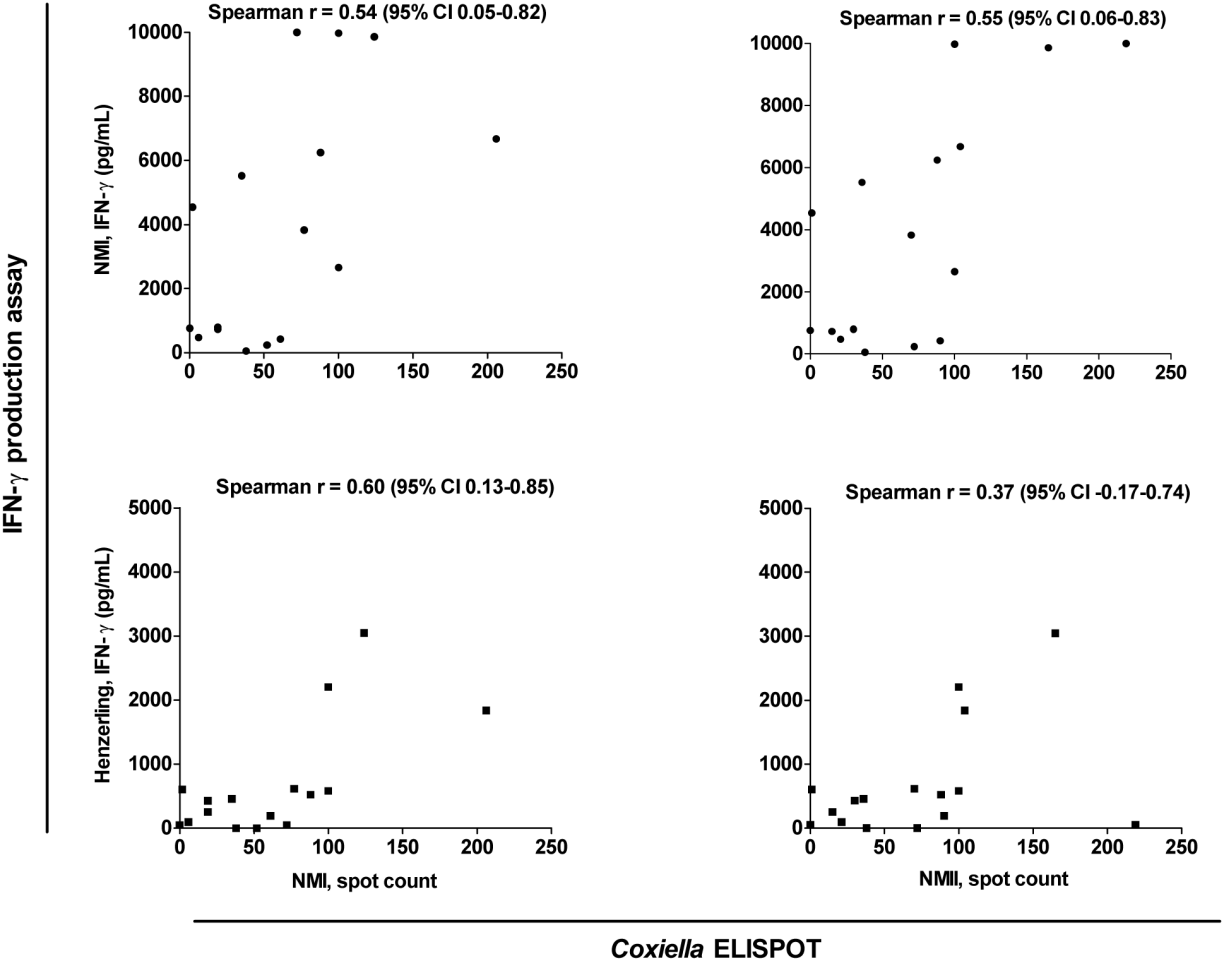
Correlation between the amount of *C. burnetii*-specific IFN-γ production and the number of IFN-γ positive cells. The individual values of chronic Q fever patients (n = 16) were used to determine the correlation between the IFN-γ production as measured with the IFN-γ production assay, and the number of IFN-γ positive cells as measured with the *Coxiella* ELISPOT. Each graph shows the correlation between the resulting values of the two assays, with either one of the stimulating antigens. On the Y-axes, the IFN-γ production is shown after stimulation with NMI (upper graphs) and Henzerling antigens (lower graphs). On the X-axes, the spot count is shown after stimulation with NMI (left graphs) and NMII (right graphs) antigens. The Spearman’s correlation coefficient *r* (95% Confidence Interval) is given for each comparison. Abbreviations: NMI, Nine Mile phase 1; NMII, Nine Mile phase 2; IFN-γ, interferon-gamma.

In the patient samples, we determined the correlation between the amount of IFN-γ produced in the IFN-γ production assay and the number of IFN-γ-producing cells in the *Coxiella* ELISPOT (Figure 3). The correlation between the values obtained in the two assays, each employing two stimulating antigens, was determined separately, resulting in a total of four comparisons. The Spearman correlation coefficient *r* ranged between 0.37–0.60, indicating a moderate to strong correlation. The discrepancies between both types of assays can be explained by the different origins and concentrations of stimulating antigens, the different phase variation, or, as mentioned previously, the method of inactivation of the antigen stimuli. Moreover, it may well be true that the amount of IFN-γ released is not directly related to the number of IFN-γ positive cells. IFN-γ based assays are used for detection of immunity to *Mycobacterium tuberculosis* and commercial kits are available for the two types of assays, e.g. the QuantiFERON-TB and the T Spot TB [14]. These assays have been reported to be at least as accurate as the tuberculin skin test to detect exposure to *M. tuberculosis*. The correlation between the two types of IFN-γ based assays for tuberculosis as previously reported in the literature, are better (*r* = 0.69 and *r* = 0.80) than what we observed evaluating these diagnostic platforms in *C. burnetii* infection [15], [16].

In conclusion, the two IFN-γ based assays had a similarly high sensitivity for detecting *C. burnetii* infection and the correlation between both test was moderate. The IFN-γ production assay in whole blood has the practical advantage of relative technical simplicity over the more laboriously intensive *Coxiella* ELISPOT. The *Coxiella* ELISPOT, however, seemed to be more specific with Nine Mile as an antigen stimulus. These observations, and previous studies of these IFN-γ based assays in *C. burnetii* infection, show the potential of measuring cell-mediated immune response in Q fever and warrant further investigation of these assays in larger cohorts of Q fever patients.
